# Performance with an additional load: formula-based predictions for controlling the load intensity when carrying backpacks

**DOI:** 10.1186/s13102-025-01111-8

**Published:** 2025-04-04

**Authors:** Saskia Klughardt, Bettina Schaar

**Affiliations:** Institute for Sports Science, Faculty of Humanity, University of the Bundeswehr Munich, Werner-Heisenberg-Weg 39, Neubiberg Munich, 85577 Germany

**Keywords:** Load carriage, Mountaineering, Military, Load intensity, Backpack, Formula, Performance, Prevention

## Abstract

**Introduction:**

Endurance-specific activities in diverse terrains, including alpine regions, necessitate the transportation of supplementary equipment, thereby necessitating an adaptation of the load intensity. To ascertain the impact of these loads on acute endurance performance and load intensity, it was essential to conduct tests with additional loads to predict the individual reaction to carrying additional loads on performance. The formulas derived in this study facilitate the prediction of exercise adaptation when carrying additional loads.

**Purpose:**

This study aimed to develop and validate a formula-based prediction of performance adaptation when carrying additional loads to guide load intensities and training instructions.

**Methods:**

The 105 participants, 54 male and 51 female, had a mean age of 23.7 years, a mean height of 174.0 cm, a mean weight of 71.7 kg, and an aerobic capacity of 48.6 mL/kg/min-1. Two treadmill ramp tests were conducted in a laboratory setting, with and without additional loads, to assess the adaptation of cardiopulmonary parameters. Both tests were conducted at 4 km/h and an incline of 1%, with the speed increasing by 1 km/h each minute until the subject reported feeling exhausted. The statistical analysis was conducted via stepwise linear regression. The formulas were validated with an independent t-test on an additional dataset, and the equivalence was determined with a two-sided test (TOST).

**Results:**

Based on these tests, regressions were calculated for speed (*p* < 0.001) and heart rate (*p* < 0.001) with additional loads, and formulas were derived to predict the adaptations of heart rate and speed to additional loads. The results revealed that the backpack weight, sex, and individual parameters without load were the most accurate predictors of performance with additional load carriage (*p* < 0.001). The validation of the formulas, using a sample of *N* = 64, was statistically equivalent.

**Conclusion:**

The formulas can predict the adaptation of running speeds and heart rates at the ventilatory thresholds with different additional loads. This is useful for controlling optimal load intensities in endurance performance with additional loads, to prevent overstraining. This is particularly relevant in mountain sports or military marches, where optimizing loads and mitigating falls due to overstraining is crucial.

## Introduction

The physical demands of hiking and mountaineering, including carrying appropriate equipment, food, and protective clothing [1], are similar to those required by the military, where physical fitness is essential for completing marches and daily duties. Nonetheless, no standardized criteria or directives exist regarding the weight of additional loads and gear in mountaineering and alpinism, nor are there mandates concerning individual fitness prerequisites. Research concerning adaptation to various additional loads resides within military contexts [2–11] or among various rescue teams, albeit without explicit recommendations regarding load intensity adjustments during performance with additional load or training protocols to prepare for load-bearing activities. Within these professional cohorts, trekking and running with additional loads are integral to daily operations, necessitating a certain level of endurance. In contrast to recreational sports contexts, military establishments, for instance, establish guidelines concerning physical performance benchmarks and requisite gear. The aggregate weight of the additional load is determined by the materials and equipment utilized, irrespective of the carrying individual's load capacity. Additionally, adherence to load specifications concerning speed or walking duration is imperative, ensuring that the additional load facilitates reaching the destination in the shortest time possible. Backpacks and equipment are typically packed based on the required gear rather than individual sex or fitness levels. Consequently, women and individuals of petite stature often bear heavier loads relative to their body mass, necessitating a higher fitness level to achieve performance comparable to that of their larger counterparts [12–14]. A need exists to examine participants with and without additional loads, assessing adaptations to delineate performance metrics when carrying additional loads. Experimental investigations involving escalating performance levels revealed that participants achieved aerobic and anaerobic thresholds with diminished cardiopulmonary metrics and performance outcomes [4, 10, 12]. Furthermore, subjective exhaustion is reported at lower performance thresholds [4, 10, 12]. Prior research has demonstrated that continuous endurance activities necessitate more time when undertaken with additional loads, as individuals engage in these activities at reduced speeds [8]. Augmented heart rate and oxygen uptake levels have been documented during predetermined tasks [7, 14, 15], indicating heightened exertion at comparable intensities. A good level of aerobic endurance can compensate for an additional load of up to 30% of the body mass if combined with a reduction in the intensity of the load [12]. The aerobic threshold has been defined in prior studies as a metric of the optimal intensity of exertion when wearing backpacks, to sustain this over an extended period [12]. Consequently, regular aerobic endurance training is recommended as a preparatory measure for handling additional loads in prolonged endurance activities [12, 15]. The evaluation of the participants under both conditions (without and with additional load) is currently essential to assess the individual adaptation depending on the additional load carried on the performance and to derive load intensities for continuous endurance performances. It is hypothesized that the expected reduction in performance when carrying additional loads can be predicted using formulas that include specific cardiopulmonary parameters at ventilatory threshold 1 (VT1) and ventilatory threshold 2 (VT2). The objective is to determine the exercise intensity and training parameters for exercise with additional load.

## Methods

### Experimental design

**Fig. 1:**
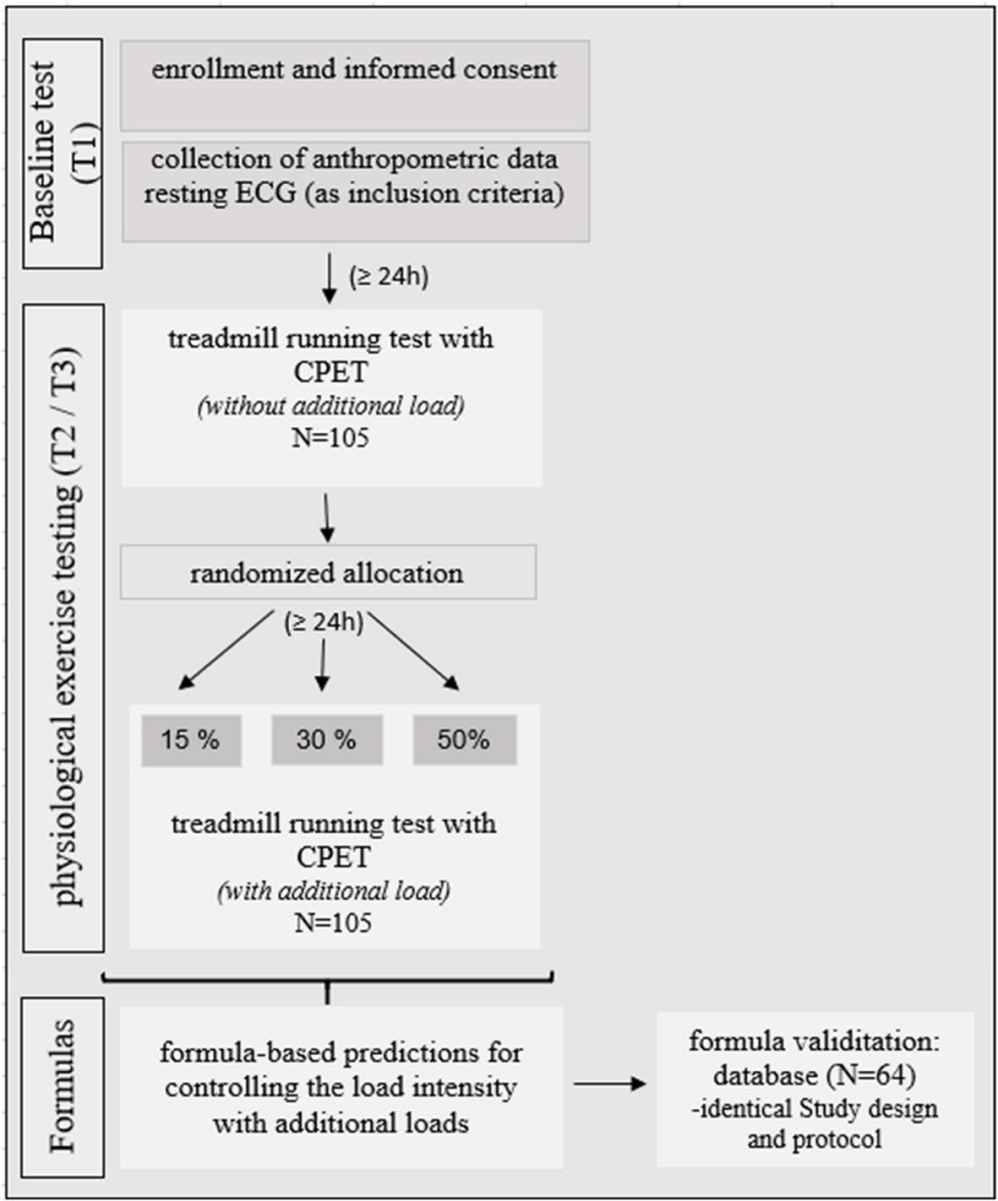
Study design

The study was conceived in an experimental design with three examination times, a baseline test (T1), and two identical, standardized treadmill tests (T2, T3) under laboratory conditions (Fig. 1). Participants were randomly assigned to the test groups according to gender. To control for confounding variables associated with the study, the same backpack (Berghaus Atlas, without removable side pockets) in small and medium sizes was used for all studies and packed with standardized weights. During the baseline test (T1), subjects were requested to complete the requisite medical and family history forms. Their height and body mass were measured, and a bioimpedance analysis (Biosen i8 Touch Maltron; UK) was performed to determine their anthropometric data. Concurrently, an electrocardiogram (ECG; Cardio 300; CustoMed; D) was conducted as a preliminary examination and inclusion criterion for participation in the treadmill tests, ensuring that the study participants possessed adequate exercise capacity. The data collated during the resting ECG were excluded from the analysis. All tests were conducted under the principles of standardized laboratory conditions. The two treadmill tests were performed at analogous times, to control the circadian fluctuations. For the initial visit, the participants were instructed to abstain from vigorous exercise and visiting the sauna for a period exceeding 24 h and to refrain from consuming food, coffee, or alcohol for a duration no longer than 12 h before the measured anthropometric data. For the subsequent two treadmill tests, the participants were requested to abstain from intensive physical activity and visit the sauna for no longer than 24 h, to ensure that participating in the tests was characterized by adequate rest and minimal pre-tiredness. During the second visit (T2), participants underwent a baseline running test in a ramp protocol with cardiopulmonary exercise testing (CPET; Metalyzer 3B-R2; Cortex-Medical; D) on a motorized treadmill (Quasar by HP Cosmos) without an additional load to determine the ventilatory thresholds (VT1, VT2), the aerobic capacity, and the subjective exhaustion by determining the VO_2_ peak. The experiment did not extend to a maximum workload performed to the point of fulfilling the criteria for maximum oxygen uptake (VO_2_ max) as outlined by the American College of Sports Medicine (ACSM; 17). Furthermore, it was essential to ensure that all participants were thoroughly acquainted with the study design, as some had no prior experience of walking on a treadmill in boots, which could have introduced a degree of bias due to the additional load. On the third visit (T3), participants underwent a running test with the CPET with a randomized additional load of 15%, 30%, or 50% of individual body mass [12]. The participants carried an average additional load of 22.8 ± 10.7 kg (32.0 ± 14.2% of their body mass). The data obtained from these tests were subjected to statistical calculations to derive formulas that predict the load intensity adjustment when additional loads are applied. Further studies were conducted with identical study designs with different additional loads to build a comparable database (*N* = 64) of male and female participants to validate the formulas of the original database [12]. In an identical study design, an initial measurement and two treadmill tests with cardiopulmonary exercise tests were performed in a ramp protocol with and without additional loads. Figure 1 outlines the study design.

Each treadmill ramp test began with a one-minute standing phase to collect baseline data. The protocol commenced with a warm-up phase lasting 3 min at 4 km/h and a 1% incline. The treadmill speed progressively increased by 1 km/h every minute. The participants transitioned from walking to running based on their subjective judgment. The test concluded when participants reached their subjective exhaustion or could not maintain the treadmill speed. At the end of the test, the maximum cardiopulmonary parameters and maximum lactate achieved, and the subjective exertion according to Borg (RPE) were recorded. Due to the subjective experience of fatigue, 17 participants could not achieve VT2 with or without an additional load before terminating the test. Fourteen participants (15%: *n* = 1; 30%: *n* = 6; 50%: *n* = 7) could not reach VT2 with an additional load before discontinuing the test due to subjective fatigue, and six participants could not reach VT2 without an additional load. Three participants were unable to get the VT2 threshold under any condition. Following the test, the participants walked at 4 km/h for 5 min to record recovery parameters. Comparable studies have investigated the effect of carrying additional loads on endurance performance. However, these studies have sometimes used very different protocols, including continuous loading at constant speed and various inclines. This has resulted in a lack of comparable results under lab and field conditions. Furthermore, it should be noted that the ventilatory thresholds are not always fully determined (6; 11; 23). There are no recommendations regarding the influence of an increasing treadmill incline on reaching the aerobic and anaerobic thresholds at a given speed. Therefore, it is essential to employ a constant incline and change in speed to investigate the influence of running speed on adaptation at different additional loads to enable further investigations to concretize the influence of the incline. Accordingly, a protocol with a constant incline was selected to investigate the impact of running speed on endurance performance. All tests were performed simultaneously each day, and the participants wore hiking boots and standard training attire, such as shorts, T-shirts, and socks. To prevent ankle injuries, the participants were required to wear hiking boots during the tests involving additional loads. These loads were aligned with the footwear typically worn in mountaineering or military marches. All participants utilized the same backpack (Berghaus Atlas II) of appropriate size, packed uniformly, and weighed accurately. The participants were equipped with a heart rate chest belt (Polar H7, FIN) and a specialized breathing mask connected to a laboratory metabolic measurement system (Metalyzer 3B-R2, Cortex-Medical; D) capable of measuring oxygen uptake (VO_2_) and carbon dioxide production (VCO_2_).

### Participants

One hundred and five healthy participants (54 men, 51 women) with a mean age of 23.7 ± 2.9 years were enrolled in this study. All participants were active military personnel aged 18 to 35 who were required to complete a 6 km annual march carrying an additional load of 15 kg within one hour. So, they are accustomed to carrying backpacks routinely. The participants had an average height of 174.0 cm ± 8.8 cm; an average body mass of 71.1 kg ± 9.9 kg; an average body fat percentage of 17.2 kg ± 4.8% of body mass; and an average aerobic capacity of 48.6 ml/kg/min-1 ± 6.9 ml/kg/min-1. Inclusion criteria were that the subjects were considered to be of normal weight and thus had a normal body mass and body fat percentage according to the ACSM standards for body composition [16]. Body fat % was used to ensure that individuals with higher muscle mass were not excluded due to an increased BMI. In addition, subjects were required to exercise regularly (8.4 ± 4.7 h per week on average) according to the ACSM and American Heart Association (AHA) recommendations for physical activity [16]. The study was conducted according to the Declaration of Helsinki guidelines and approved by the University of the Bundeswehr ethics committee in Munich. Before voluntary participation, participants were provided with information regarding the study's objectives and potential risks, and they provided written informed consent. The research project and all associated documents were submitted for review by the ethics committee and the data protection officer of the University of the Bundeswehr in Munich. Following this review, the project was subsequently approved.

### Cardiopulmonary exercise testing measurements

Before the commencement of the experimental trials, a preliminary warm-up period of greater than 45 min was initiated for the metabolic system. After this, the flowmeter and the two gas analysers were calibrated under the manufacturer's guidelines before each test. Post-processing of the data obtained with the software 'Meta Soft Studio' (Cortex-Medical; D) was performed with this software and prepared for further statistical analysis. This process entails the retrospective identification of outliers within the dataset, followed by calculating moving averages for 15 breaths [17, 18]. The evaluation of VT1 was subsequently conducted via the V-slope method [19] involving the VE/VO_2_ curve and the PETO_2_ curve. VT2 is determined via the ratio of minute ventilation to carbon dioxide production (the VE/VCO_2_ slope), the breathing equivalent for carbon dioxide, and the PETCO_2_. A combination of shape analysis and systematic evaluation has become the standard approach for determining ventilatory thresholds, and the reliability of this method is considered acceptable. Maximum/Peak oxygen uptake is determined over a 30-s interval at the end of exercise [17–20]. The exercise was intentionally terminated when the subjects reported subjective exhaustion, rather than continuing until a “levelling off” was observed. As a result, a VO_2_ peak rather than a VO_2_ max was recorded.

### Outcome variables

The analysis examined the following parameters to ascertain the factors affecting speed and heart rate adaptation under additional loads: participant speed (km/h), heart rate (bpm), and VO_2_ (L/min) reached at VT1 and VT2, both with and without additional loads. Additionally, factors such as sex (male, female), backpack weight (% / kg), height (cm), body mass (kg), body fat (%), physical activity (h/week), maximum heart rate (bpm), and relative maximum oxygen uptake (mL/kg/min-1), maximum lactate (mmol/L) and RER were considered.

### Statistical analysis

A stepwise selection process was employed from a comprehensive set of variables to identify the most effective predictors for a linear model assessing speed and heart rate adaptation when carrying additional loads at VT1 and VT2. These variables included sex, cardiopulmonary parameters at VT1 and VT2, subjective exhaustion, and body composition. The initial step was to ascertain whether multiple linear regression prerequisites were fulfilled. The model demonstrates the linearity of the variables, the absence of outliers, and the absence of autocorrelation, as evidenced by the value of the Durbin‒Watson statistic, which is ~ 2. The values of tolerance, > 0.1, and VIF, < 10, substantiate the absence of multicollinearity between the predictors. Moreover, the model exhibits homoscedasticity, with a value of > 0.5, and a normal distribution of the residuals, with a value of < 0.5 [21]. The stepwise regression method was implemented bidirectionally: forward selection of variables and interactions from the model without variables and backward elimination at each step after introducing a new variable or interaction. To interpret the coefficients of multiple determination R^2^, Cohen's rules of thumb were used to categorize the explanation of variance. The following categories were used: low/weak variance explanation |R^2^|= 0.02; medium/moderate variance explanation |R^2^|= 0.13; high/strong variance explanation |R^2^|= 0.26 [22]. To validate the formulas, we used a dataset of 64 participants (31 men and 33 women) with similar ages and heights (23.5 ± 2.4 years, t (167) = −0.37, *p* = 0.714; height 174.2 ± 9; *p* = 0.865) However, the anthropometric data differed, with a body mass of 74.5 ± 12.8 kg (t (167) = 1.98, *p* = 0.049) and body fat of 20.3 ± 6.3% body mass (t (167) = 3.52, *p* < 0.001). In an identical study design, the participants also underwent two treadmill tests with cardiopulmonary exercise tests in an identical ramp protocol with and without an additional load. The validation dataset underwent analysis with a comparable additional load of 25.0 ± 9.5 kg (*p* = 0.436) or an additional load measured as a percentage at 33.2 ± 10.4% of the individual body mass (*p* = 0.130). Following the assessment of the normal distribution, independent samples t-tests were conducted to ascertain potential differences between tests conducted with additional loads and parameter calculations derived from the formulas. The effect size was calculated using Cohen's 'd'. The following categories were used: small effect |d|= 0.2; medium effect |d|= 0.5; large effect |d|= 0.8 [22]. To confirm the relationship between the measurements with additional loads and the calculated results via the formulas, we determined the equivalence via two one-sided tests (TOSTs). Confirm the effect between the upper and lower limit values and the equivalence to zero by conducting both tests and obtaining significant results [23]. Cicchetti's criteria were utilized to categorize the intraclass correlation (ICC), whereby a value of < 0.4 is designated as "poor", a value ranging from 0.40–0.59 is classified as "average", a value ranging from 0.6–0.74 is categorized as "good" and a value of > 0.75 is labeled as "very good" [24]. The statistical analyses were conducted using SPSS (version 29), provided by the University of the Bundeswehr in Munich. A significance level of 0.05 was employed to determine statistical significance. An asterisk (*) is used to indicate results of *p* < 0.001 that are deemed to be highly significant.

## Results

Table 1 presents the mean, standard deviation, minimum, and maximum values for each variable included in this study.

**Table 1:** Means, standard deviations, and values for each variable included in the statistical calculations

Variable	Without additional load	With additional load 32.0 ± 14,2%	*p*-value
Speed (km/h) at VT1	9.3 ± 1.4	7.4 ± 1.2	< 0.001*
HR (bpm) at VT1	157 ± 14	152 ± 16	0.002
VO_2_ (L/min) at VT1	2.4 ± 0.6	2.3 ± 0.6	< 0.001*
Speed (km/h) at VT2	12.9 ± 1.8	10.0 ± 1.8	< 0.001*
HR (bpm) at VT2	182 ± 10	176 ± 10	< 0.001*
VO_2_ (L/min) at VT2	3.2 ± 0.7	3.0 ± 0.7	< 0.001*
HR peak (bpm)	187 ± 10	183 ± 9	< 0.001*
VO_2_ peak (mL/kg/min-1)	48.6 ± 6.9	46.9 ± 7.2	< 0.001*

Data are presented in mean ± SD. *N* = 105 (VT2; *N* = 88)

*bpm beats per minute* km/h kilometres per hour, *L/min* litres per minute, *mL/kg/min-1* millilitres per kilogram per minute, *SD* standard deviation, *VO*_2_ oxygen uptake, *peak* value at peak exercise, *VT1* ventilatory threshold 1, *VT2* ventilatory threshold 2

**Fig. 2:**
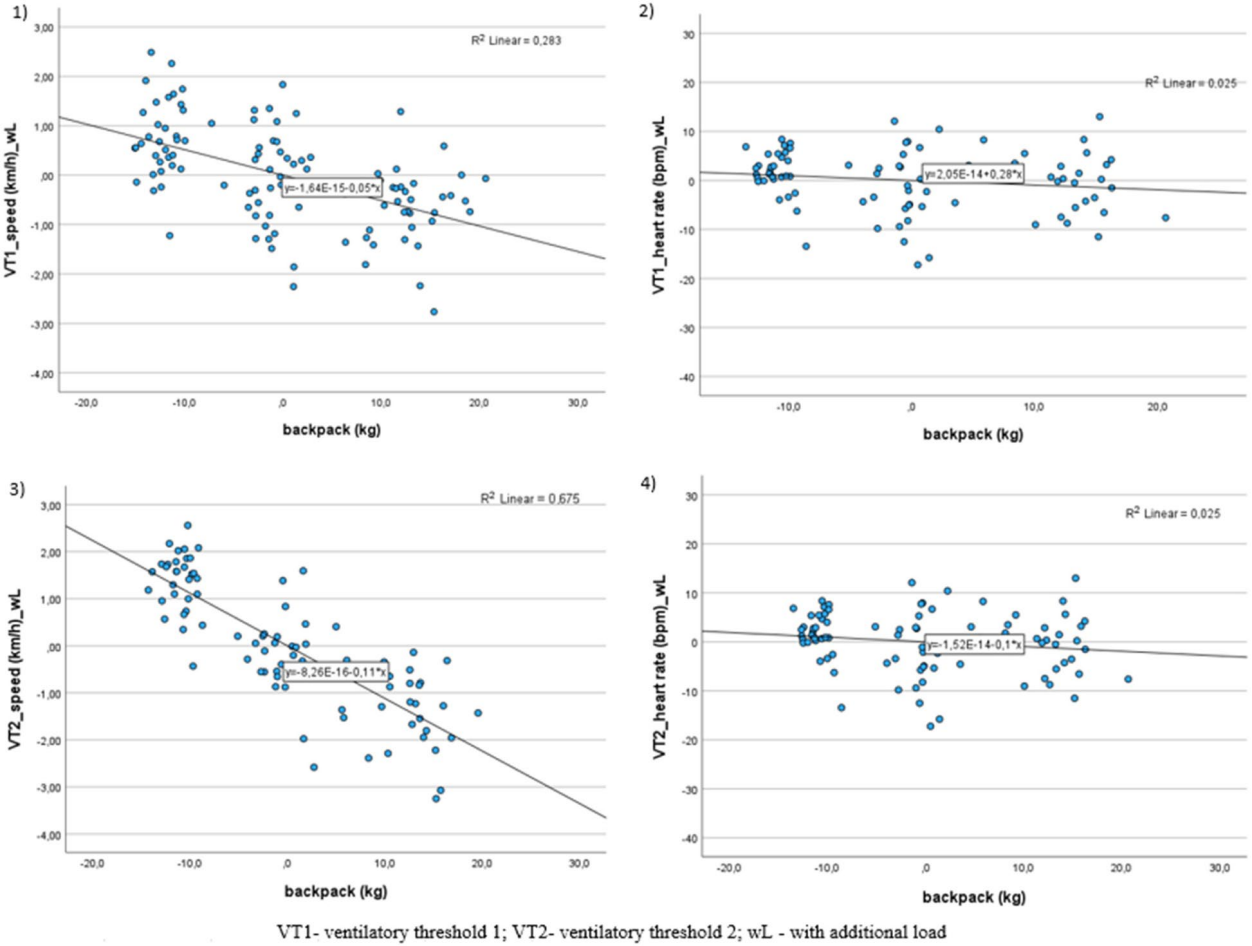
Plots of regression curves to visualize the relationship between backpack weight, speed, and heart rate at the ventilatory thresholds (VT1, VT2) with additional loads (wL)

In the regression analysis, the sample size was 88 subjects, as 17 participants did not reach VT2 and were excluded from the calculation. Fourteen participants could not reach VT2 with an additional load (15%: *n* = 1; 30%: *n* = 6; 50%: *n* = 7) before discontinuing the test due to subjective fatigue, and six participants could not reach VT2 without an additional load. Of these participants, 3 could not reach VT2 with or without additional load. Regarding the stepwise analysis results, the most effective predictors for adapting speed and heart rate when carrying additional loads are the parameters without additional load, backpack weight, sex, and their interaction. Figure 2 shows the relationship between the backpack load in kilograms and parameters speed and heart rate at the ventilatory thresholds.

The model demonstrated a high goodness of fit for the parameters at VT1, with an R-squared value of 0.49 (adjusted R-squared = 0.47) for speed, and a moderate goodness of fit, with an R-squared value of 0.38 (adjusted R-squared = 0.36) for heart rate [22]. The model demonstrated high goodness of fit for speed at VT2, with an R-squared value of 0.82 (adjusted R-squared = 0.82), and for heart rate, with an R-squared value of 0.65 (adjusted R-squared = 0.63) [22]. Table 2 presents the estimated parameters and the resulting speed and heart rate model with additional loads. The predictor's height (in cm), body mass (in kg), HR peak (bpm), VO_2_ (in L/min) at the ventilatory thresholds, and relative VO_2_ peak (mL/kg/min-1) were not statistically significant, indicating that this variable exerts only a minor influence on adaptation with an additional load on VT1 and VT2 and is therefore poorly suited for prediction. The changes in the adjusted R-squared values resulting from the stepwise regression were interpreted as indicators of the percentage of variance in the adaptation of speed and heart rate when carrying additional loads, explained by the predictors incorporated into the model.

**Table 2:** Estimated parameters and their significance in the resulting model for speed and heart rate with additional loads

Parameter	Estimate	SE	t	p
**VT1 (N = 105)**				
**Speed (km/h) at VT1**				
Intercept	4.85	0.78	6.21	< 0.001*
Sex	−0.32	0.18	−1.72	0.088
Additional load (kg)	−0.05	0.01	−6.31	< 0.001*
Speed (km/h) at VT1	0.45	0.07	6.88	< 0.001*
R^2^ = 0.485; F (3,101): 31.96, *p* < 0.001				
**Heart rate (bpm) at VT1**				
Intercept	30.77	15.51	1.98	0.050
Sex	3.25	2.68	1.21	0.228
Additional load (kg)	0.28	0.12	2.28	0.025
Speed (km/h) at VT1	0.70	0.10	7.32	< 0.001*
R^2^ = 0.381; F (3,101): 20.72, *p* < 0.001				
**VT2 (*N*** **= 88)**				
**Speed (km/h) at VT2**				
Intercept	4.71	1.09	4.31	< 0.001*
Sex	−0.66	0.20	−3.28	0.001
Additional load (kg)	−0.11	0.01	−13.22	< 0.001*
Speed (km/h) at VT2	0.69	0.07	10.11	< 0.001*
R^2^ = 0.822; F (3,101): 129.42, *p* < 0.001				
**Heart rate (bpm) at VT2**				
Intercept	26.78	13.40	2.00	0.049
Sex	−2.24	1.32	−1.69	0.095
Additional load (kg)	−0.10	0.07	−1.45	0,150
Speed (km/h) at VT2	0.86	0.07	11.98	< 0.001*
R^2^ = 0.645; F (3,101): 50.91, *p* < 0.001				

*SE *standard error, *sex* 1 for male, 2 for female, *VT1* ventilatory threshold 1, *VT2* ventilatory threshold 2

The regression formulas for predicting the adaptation of speed and heart rate at VT1 and VT2 when carrying additional loads are presented in the following four formulas (formulas 1, 2, 3 and 4):

Formulas for the parameters at ventilatory threshold 1 (VT1).

1 $$\mathrm{Speed}\;\mathrm{at}\;\mathrm{VT}1\;\mathrm{with}\;{\mathrm{backpack}}_{\;\left(\mathrm{km}/\mathrm h\right)}\;=\;4.853\;-\;0.316\ast\;{\mathrm{sex}}_{\;\left(1\;\mathrm{for}\;\mathrm{males};\;2\;\mathrm{for}\;\mathrm{females}\right)}\;+\;0.449\ast\;\mathrm{speed}\;{(\mathrm{VT}1)}_{\;\left(\mathrm{km}/\mathrm h\right)}\;-\;0.052\ast\;\mathrm{backpack}\;{\mathrm{weight}}_{\left(\mathrm{kg}\right)}$$

2 $$\mathrm{Heart}\;\mathrm{rate}\;\mathrm{at}\;\mathrm{VT}1\;\mathrm{with}\;{\mathrm{backpack}}_{\left(\mathrm{bpm}\right)}\;=\;30.766\;+\;3.250\ast\;{\mathrm{sex}}_{\left(1\;\mathrm{for}\;\mathrm{males};\;2\;\mathrm{for}\;\mathrm{females}\right)}\;+\;0.704\ast\;\mathrm{HR}{(\mathrm{VT}1)}_{\left(\mathrm{bpm}\right)}\;\;+\;0.281\ast\;\mathrm{backpack}\;{\mathrm{weight}}_{\left(\mathrm{kg}\right)}$$

Formulas for the parameters at ventilatory threshold 2 (VT2).

3 $$\mathrm{Speed}\;\mathrm{at}\;\mathrm{VT}2\;\mathrm{with}\;{\mathrm{backpack}}_{\left(\mathrm{km}/\mathrm h\right)}\;=\;4.714\;-\;0.655\ast\;{\mathrm{sex}}_{(1\;\mathrm{for}\;\mathrm{males};\;2\;\mathrm{for}\;\mathrm{females})}\;+\;0.686\ast\;\mathrm{speed}\;{(\mathrm{VT}2)}_{(\mathrm{km}/\mathrm h)}\;-\;0.112\ast\;\mathrm{backpack}\;{\mathrm{weight}}_{(\mathrm{kg})}$$

4 $$\mathrm{Heart}\;\mathrm{rate}\;\mathrm{at}\;\mathrm{VT}2\;\mathrm{with}\;{\mathrm{backpack}}_{(\mathrm{bpm})}\;=\;26.777\;-\;2.237\ast\;{\mathrm{sex}}_{(1\;\mathrm{for}\;\mathrm{males};\;2\;\mathrm{for}\;\mathrm{females})}\;+\;0.856\ast\;\mathrm{HR}{(\mathrm{VT}2)}_{(\mathrm{bpm})}\;-\;0.096\ast\;\mathrm{backpack}\;{\mathrm{weight}}_{(\mathrm{kg})}$$

No significant differences (*p* > 0.05) were observed between the data obtained with the additional load and the heart rate and speed values calculated via the formulas. Table 3 presents the validity of the formulas concerning a further database with additional loads.

**Table 3:** Validity of the formulas concerning a further sample in tests

	**Ramp test (*N*** **= 64)**	**Formula (*N*** **= 64)**			
	Mean ± SD	Mean ± SD	t	p	d
Speed (km/h) at VT1	6.7 ± 0.9	6.9 ± 0.6	−1.8	0.082	−0.22
Heart rate (bpm) at VT1	149 ± 16	151 ± 11	−1.3	0.192	−0.17
Speed (km/h) at VT2	9.1 ± 1.2	9.3 ± 1.0	−1.6	0.107	−0.21
Heart rate (bpm) at VT2	174 ± 9	175 ± 9	−0.9	0.379	−0.11

*SD* standard deviation, *VT1* ventilatory threshold 1, *VT2* ventilatory threshold 2, *d Cohen’s d* effect size

The formulas for calculating speed confirm significant results at VT1 and VT2 in both tests, indicating that they are equivalent to zero. Concerning the two formulas for heart rate at VT1 and VT2, the TOST test did not yield significant results. The formulas for calculating speed confirm significant results at VT1 and VT2 in both tests, indicating that they are equivalent to zero. Concerning the two formulas for heart rate at VT1 and VT2, the TOST test did not yield significant results The results of the TOST test, which assesses the equivalence of the results in Table 3, are shown in Table 4.

**Table 4:** Test of equivalence (TOST) to compare the formulas versus tests with additional load

		**t**	**df**	**p**	**d**	**95% CI**
Speed (km/h) at VT1	t-test	1.77	63	0.082		
	TOST Lower	6.19	63	< 0.001*	-0.55	-0.06
	TOST Upper	-0.27	63	0.005	0.55	0.51
Heart rate (bpm) at VT1	t-test	1.32	63	0.192		
	TOST Lower	1.62	63	0.055	-0.38	-0.07
	TOST Upper	1.02	63	0.844	0.38	0.40
Speed (km/h) at VT2	t-test	1.64	60	0.107		
	TOST Lower	5.66	60	< 0.001*	-0.52	0.00
	TOST Upper	-2.39	60	0.01	0.52	0.43
Heart rate (bpm) at VT2	t-test	0.89	60	0.379		
	TOST Lower	1.34	60	0.093	-0.06	-0.11
	TOST Upper	0.43	60	0.667	0.06	0.34

*TOST* two one-sided tests, *VT1* ventilatory threshold 1, *VT2* ventilatory threshold 2, *d Cohen’s d* effect size

The intraclass correlation (ICC) was used to estimate the strength of the correlation and assess the reliability of the formulae for the data obtained with the additional loading of the validation dataset. The intraclass correlation (ICC) for speed at VT1 (0.458) was considered average and the parameters heart rate at VT1 (0.684) were considered good, the speed at VT2 (0.776) and heart rate at VT2 (0.752) were considered very good [24]. The results of the ICC test, are shown in Table 5.

**Table 5:** Test of intraclass correlation (ICC) to compare the formulas versus tests with additional load

	**N**	**Formula**	**Database**	**ICC**	**95% CI**
Speed (km/h) at VT1	64	6.92 ± 0.60	6.72 ± 0.92	0.486	[0.165; 0.686]
Heart rate (bpm) at VT1	64	151 ± 11	149 ± 16	0.684	[0.482; 0.808]
Speed (km/h) at VT2	64	9.31 ± 1.00	9.11 ± 1.24	0.766	[0.612; 0.859]
Heart rate (bpm) at VT2	64	175 ± 9	174 ± 11	0.752	[0.588; 0.851]

*ICC *intraclass correlation, *VT1* ventilatory threshold 1, *VT2* ventilatory threshold 2

## Discussion

The current body of research utilizes supplementary loads ranging from 1 to 3 kg in specially designed running backpacks [25] and up to 45 kg [3]. The present study incorporated a maximum supplementary load of 45.9 kg, equivalent to 50% of the individual's body mass [12]. Nonetheless, classifying supplementary loads based on kilograms fails to consider the distinctive physical attributes of the test subjects. A systematic review by [13] already posits that the influence of additional loads is comparable when they are selected proportionate to the body constitution. Furthermore, research findings have already demonstrated that weight loads of 15 to 20 percent of body mass are recommended for adults and 13 to 15 percent for young adults [26]. These additional loads can be readily incorporated into recreational and health sports. However, in professional contexts such as the military or alpine tours and expeditions, the necessity of carrying additional equipment, attire changes, cold/wet protection, and provisions, can lead to total loads that exceed the recommended 15 to 20% of body mass. It can be posited that the selection of additional loads of 15%, 30%, or 50% of the individual body mass of the participants was adequate for the generation of a comparable load and stress and the coverage of a broad spectrum of additional loads. The fundamental study on these formulas demonstrates that carrying additional loads is associated with a reduction in running speed of up to −36% in men and −32% in women [12], which is proportional to the amount of additional load being carried. The aerobic threshold is the intensity of exertion required to sustain exertion with additional loads over a longer time [12]. The existing recommendations for mountain sports [26], which are based on performance with additional loads of 15 to 20% of body mass for adults, can be further extended by the results of this study. For physically active individuals, additional loads of up to 30% of the individual's body mass are tolerable if the aerobic threshold is used as a measure of load intensity [12].

This study revealed that formulas for reducing speed while carrying an additional load apply to males and females aged 18 to 35, irrespective of their body composition. The reduction in performance when carrying additional loads can be calculated prospectively based on formulas using the parameters of unloaded speed, sex, and weight of the backpack at the aerobic and anaerobic thresholds to derive load intensities in terms of speed as parameters for performance with additional loads. These formulas can predict reductions in speed performance on the treadmill with additional loads, without needing a test with additional loads. In the context of treadmill testing, which involves the application of an augmented load, there exists a potential for overloading and consequent injury. Using established formulas can serve as a means to mitigate this risk. However, in contexts such as mountain sports or the military, where the routine involves the carriage of additional loads, there is a necessity for information regarding the influence of diverse loads on performance. The formulas for heart rate adaptation did not result in an effect close to zero [23], as shown in Table 2, where the standard deviation in heart rates at VT1 and VT2 is high. Heart rate is highly individualized based on training type and level, making comparisons difficult. Women reach the aerobic threshold at comparable heart rates with 30 to 50% additional load, whereas men at lower heart rates have 15 to 30% additional load [12].

The findings of the study on which the formulae are based demonstrated that women attain the anaerobic threshold at a lower heart rate with 15 to 50% additional load than in the absence of load, concurrently with men reaching the anaerobic threshold with 15 and 50% additional load at a comparable heart rate [12]. Therefore, there is no clear trend in the heart rate with and without additional load, which is also reflected in the formulas. Before developing the formulas, it was imperative to test both with and without additional loads to predict the performance and load reduction due to additional loads and to obtain concrete results, indicating that, solely in the case of the additional load that was tested, a prediction could be made as to what performance could have been achieved with this additional load to avoid overloading. The formulae can now be used to predict an adjustment of the individual load intensity with different additional loads when running on a treadmill without an incline. Further studies in field conditions, incorporating diverse terrain types, are required to ascertain the applicability of these formulas in varied terrain and field settings.

In addition, the aerobic threshold is a benchmark for the intensity of the load when carrying additional loads, as higher intensities lead to a disproportionate increase in stress concerning the level of the additional load and consequently increase the risk of overstressing as well as falls and accidents [12]. However, this is only occasionally used as a parameter for carrying additional loads [10, 12, 14]. For the assessment of aerobic capacity, aerobic and anaerobic thresholds are critical submaximal endurance parameters [18]. Assessing endurance performance with additional loads plays a crucial role in mountain sport-specific endurance performance, such as hiking and mountaineering, and in an occupational context, such as the military, between both sexes [24, 27]. Studies with additional loads rarely consider aerobic and anaerobic thresholds [10, 12, 14]. Previous studies have consistently demonstrated that heart rate and VO_2_ increase during continuous performance with absolute additional loads, regardless of the load intensity [4, 6, 27]. Even with light additional loads of 1 to 3 kg, such as those used in trail running, increases in heart rate and VO_2_ can be shown at a performance intensity of 70 to 90% of the aerobic capacity [25] if not adjusted for individual body composition. The categorization of additional loads in kilograms, not considering individual performance capacity and individual physical constitution, means that it is impossible to compare the load caused by the additional load and the possible performance when carrying it [12, 13]. This is crucial for group performance and as an assessment criterion in settings where carrying an additional load is mandatory to determine the training modalities and intensities that can be achieved with the additional load without provoking overexertion. A group of mountaineers' performance capacity is limited by the participant with the lowest relative performance [28]. The backpack weight in kilograms and the individual aerobic capacity are critical criteria for determining the appropriate load intensity when carrying additional loads to achieve optimal continuous performance and reduce the risk of overexertion.

Sex did not significantly affect the speed at VT1 or the heart rate at VT1 or VT2. Nevertheless, it significantly affects the speed at VT2 with additional loads (*p* < 0.001). However, women experience more stress than men during standardized performance, with a comparable backpack weight (in kg) at the VT2 speed [14]. This confirms that sex plays a significant role in the higher performance of individuals under additional loads [14]. Additionally, the study establishes sex as a predictor.

Body-Mass-Index and maximum oxygen uptake have been reliable predictors of performance in obstacle courses with additional loads in military settings [11]. The findings of the study on which the formulae are based also demonstrate that the relative VO_2_ max exhibits a highly significant correlation (*p *> 0.001*) with the speed achieved at VT1 and VT2 [12]. It has been demonstrated that a high aerobic capacity, as defined by the ACSM [16], is inadequate for exercise involving additional loads of ≥ 30% of the individual's body mass. This necessitates cardiorespiratory fitness that exceeds the norm, as defined by the ASCM with a VO_2_ max of 55–56 mi/min/kg [16], to counteract the substantial performance losses that result from carrying high additional loads of 50% of body mass [11]. VO_2_ max is crucial for assessing maximum cardiopulmonary fitness [16]. For additional loads exceeding 50% of body mass, exceptional cardiopulmonary fitness is needed, as measured by the relative VO_2_ max. It corresponds to a VO_2_ max of over 46 mL/kg/min-1 for women and over 57 mL/kg/min-1 for men [12, 16]. Despite the high influence of relative VO_2_ max on the reduction of exercise capacity when carrying additional loads [12], relative VO_2_ max did not yield significant results as a predictor in the regression models (*p* > 0.5). Therefore, it had no additional value as a predictor of exercise capacity reduction and reduced the goodness of fit for the parameters at VT1 and VT2, with a lower R-squared. Accordingly, relative VO_2_ max was excluded as a predictor. Consequently, the formulas could not be substantiated with maximum parameters such as HRmax and VO_2_ max, as these did not yield significant results and are, therefore, not meaningful predictors. The exercise was intentionally stopped at subjective exhaustion rather than continuing to levelling off as a VO_2_ max benchmark [29, 30]. Although the subjects achieved an RER of 1.10 ± 0.05, an RPE of 18.7 ± 1.2 and a maximum lactate of 8.4 ± 2.1 mmol/L, VO_2_ plateauing was not achieved, so absolute VO_2_ max cannot be assumed [30]. Considering VO_2_ max as a predictor would also limit the formulas to data generation via cardiopulmonary exercise testing, as this is the only way to determine VO_2_ max. In the absence of this parameter, lactate performance diagnostics can also be used to test the validity of the formulas and the transfer of aerobic and anaerobic thresholds determined by ventilatory thresholds to lactate thresholds. These formulas can be confidently used for various diagnostics, expanding their potential.

In addition, the individual load intensity and training parameters for different additional loads can be derived, which can be used for mountaineering tours, marches, and training programs to prepare for activities with additional loads. It benefits occupational groups such as the military, as carrying additional loads is routine. The precision of input findings can be enhanced by establishing a correlation between job-specific requirements, such as the weight of additional loads, and the performance that recruits bring. This correlation will facilitate the derivation of individual training recommendations. In mountain sports, adjusting the pace to the terrain is essential, considering factors such as steepness and trail conditions [31]. For the external validity of the formulas, it is necessary to test the data collected under laboratory and field conditions. The formulas are applicable for determining the speed adapted to the additional load for marches on flat terrain. For loads under irregular terrain conditions, such as ascents or changing terrain, controlling the intensity via the individual heart rate at the aerobic or anaerobic threshold is recommended to prevent overstrain.

### Limitations

The formulae were determined in a laboratory using a treadmill with a 1% incline. Further studies are required to ascertain their applicability in different terrains, with varying gradients, and under field conditions. Moreover, the present study and the resulting formulae illustrate endurance solely in the context of motor movement forms, thereby presenting it as a singular influencing factor. A further refinement of the results could be achieved by integrating biomechanical parameters within the context of gait analysis. Further research demonstrated that carrying additional loads diminished dynamically stable muscle activity and augmented activity in the upper trapezius, erector spinae, rectus femoris, and gastrocnemius medialis [32]. Moreover, walking uphill led to diminished stable muscle activation and augmented activity in the rectus femoris and gastrocnemius medialis, accompanied by alterations in the gait pattern [32]. The present study investigated the short-term effects of carrying backpacks during a standardized treadmill test. However, no results are available on the long-term impacts of frequent heavy rucksack carriage. Nevertheless, the current data set provides a solid foundation for further research, with the potential to design promising studies.

## Conclusion

The results confirm that the formulas can reliably predict the adaptation of running speeds and heart rates in VT1 and VT2 with additional loads under laboratory conditions. To demonstrate the formulas' external validity, it is essential to apply them in an athletic environment under field conditions to generate optimal load intensities when carrying additional loads and implement structured training control in preparation for carrying additional loads. On this basis, further research can extend the formulas for changing terrain conditions.

## Data Availability

The datasets used and/or analysed during the current study are available from the corresponding author upon reasonable request.

## Abbreviations

ACSM: American College of Sports Medicine
AHA: American Heart Association
CPET: Cardiopulmonary exercise test
d: Cohen´s effect size “d”
ECG: Electrocardiogram
HR max: Maximum heart rate
ICC: Intraclass correlation
PETCO_2_: Partial pressure of end‑tidal CO_2_
PETO_2_: Partial pressure of end‑tidal O_2_
RER: Respiratory exchange ratio
RPE: Rate of perceived exertion
SE: Standard error
T1: Baseline test
T2: Treadmill running test without additional load
T3: Treadmill running test with randomized additional load
TOST: Two one-sided tests
VCO_2_: Carbon dioxide uptake
VE/VCO_2_: Ventilatory equivalent for carbon dioxide
VE/VO_2_: Ventilatory equivalent for oxygen
VIF: Variance inflation factor
VO_2_: Oxygen uptake
VO_2_ max: Maximum oxygen uptakeVT1 – ventilatory threshold 1
VT2: Ventilatory threshold 2
wL: With additional loads
